# Flocculent Ti_3_AlC_2_-Based Two-Dimensional Material Flexible Microneedle Patch for Screening Alzheimer’s Disease Animal Biomarkers and In Situ Revealing Spatial Aggregation of Arachidonic Acid

**DOI:** 10.3390/molecules31203665

**Published:** 2026-10-13

**Authors:** Siyuan Chen, Bixiang Li, Tangjing Zhao, Qin Huang, Chengyu Li, Yilamujiang Ainiwan, Songtao Qi, Jun Pan

**Affiliations:** 1 Department of Neurosurgery, Nanfang Hospital, Southern Medical University, Guangzhou 510515, China; wgrasssfish@163.com (S.C.); tangjingzhao7713@163.com (T.Z.); huangqin67@outlook.com (Q.H.); chengyuli4967@163.com (C.L.); yilamujiang6809@163.com (Y.A.); 2 Department of Neurology, Zhujiang Hospital, Southern Medical University, Guangzhou 510515, China; icarusforever@163.com

**Keywords:** Alzheimer’s disease, metabolomics, flexible microneedle, Ti_3_AlC_2_, parietal lobe, arachidonic acid, spatial aggregation

## Abstract

Efficient diagnosis and pathological elucidation of Alzheimer’s disease (AD) are constrained by the lack of technologies for in situ capture of metabolites in living brain tissues. Metabolic disorders in the parietal lobe highly correlate with AD progression, yet existing methods cannot achieve in situ capture and spatial analysis. A petal-like flocculent Ti_3_AlC_2_-based flexible microneedle patch was developed for in situ extraction and mass spectrometry of parietal metabolites in AD rabbits. Sodium hydroxide etching and cetyltrimethylammonium bromide (CTAB) self-assembly constructed a flocculent micro-/nanostructure, enhancing enrichment ~4-fold (2.5-fold after etching + 1.6-fold after CTAB). The stretchable substrate enables conformal attachment to the curved parietal lobe. Application to the exposed cortex demonstrates mechanical flexibility, linear glucose response (R^2^ = 0.9958) and resistance to salt/protein interference. Serum profiling of 15 AD patients and 15 controls with OPLS-DA shows clear discrimination (top-5 markers combined area under the curve (AUC) = 0.84). Spatial analysis reveals arachidonic acid’s unique pattern of centralized aggregation with peripheral gradient diffusion. This novel in situ device enables brain metabolite detection and reveals abnormal arachidonic acid aggregation, providing evidence for lipid-pathway AD interventions.

## 1. Introduction

**Scheme 1:**
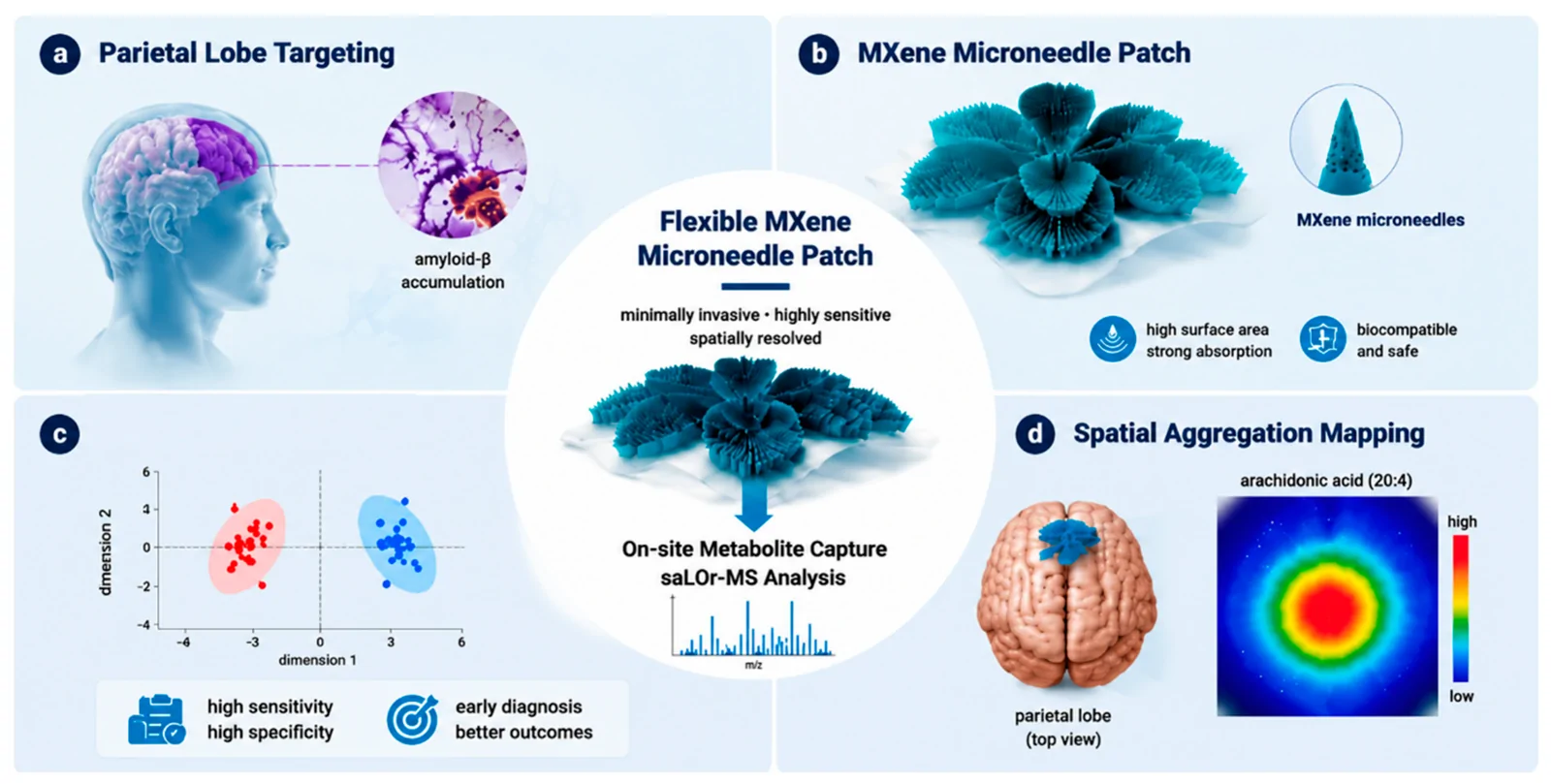
Schematic overview of the flexible MXene microneedle patch integrated with MS for spatially resolved metabolomic detection targeting parietal lobe amyloid-β lesions in Alzheimer’s disease (AD). Central panel illustrates the core workflow of the platform: in situ efficient capture of interstitial metabolites followed by MS measurement, with inherent advantages including efficient in situ sampling after craniotomy with limited additional tissue disruption, superior detection sensitivity and spatial resolution. (**a**) Pathological background of AD: massive amyloid-β aggregation specifically accumulates in the parietal lobe, the critical brain region responsible for cognitive dysfunction. (**b**) Structural illustration of the MXene microneedle patch; the porous MXene microneedle array features large specific surface area, strong molecule adsorption capacity, favorable biocompatibility and biosafety for metabolite enrichment. (**c**) Multivariate statistical discrimination based on MS metabolomic profiling, which achieves high analytical sensitivity and specificity to support efficient AD diagnosis and improve clinical therapeutic outcomes. (**d**) Spatial aggregation heatmap of arachidonic acid, visualizing the gradient distribution of parietal lobe characteristic metabolites associated with AD pathological lesions.

Alzheimer’s disease is a clinically prevalent neurodegenerative disorder characterized by progressive cognitive impairment and behavioral deficits (Scheme 1a). Its complex pathogenesis and the difficulty of efficient diagnosis impose substantial burdens on families and society [1,2,3]. The cerebral parietal lobe, as a high-level cognitive integration center, participates in spatial perception, attentional regulation, and language comprehension. Recent studies indicate that AD progression is closely associated with metabolic network dysregulation in the parietal lobe, which exhibits metabolic reprogramming—such as decreased glucose metabolism, abnormal phospholipid metabolism, and neurotransmitter imbalance—at a suitable stage [4,5,6,7]. However, existing methods (e.g., cerebrospinal fluid extraction, positron emission tomography) struggle to achieve in situ efficient capture and precise quantification of metabolites in living brain tissues. Consequently, the dynamic changes and spatial distribution characteristics of local parietal metabolites under pathological states remain unclarified, hindering the understanding of AD pathogenesis and the discovery of efficient biomarkers.

Surface-assisted laser desorption/ionization mass spectrometry (SALDI-MS) offers advantages including rapid detection, simple sample preparation, suitability for complex biofluid analysis, and efficient qualitative/quantitative determination of small-molecule metabolites, rendering it a core technology in metabolomics [8,9,10,11,12]. The analytical performance of SALDI-MS relies heavily on the matrix material. Recently, two-dimensional (2D) materials such as MXenes and graphene have shown great potential as SALDI matrices due to their large specific surface areas, excellent electron transport capacities, and laser desorption/ionization catalytic activities [13,14,15,16,17,18,19,20]. Nevertheless, when directly applied for in situ metabolite collection in living brain tissues, conventional 2D matrices face limitations such as low sampling efficiency, narrow metabolite coverage, and insufficient resistance to biological matrix interference. Moreover, traditional rigid substrates cannot conform to the curved contours of brain tissue, preventing tight attachment and stable sampling [21,22,23,24,25].

To overcome these limitations, we designed a flocculent Ti_3_AlC_2_-based 2D material flexible microneedle patch (MXene-MNs-SALDI-Biopatch) (Scheme 1b). The patch integrates three core functional modules: (1) a microneedle array structure that penetrates and adsorbs metabolites from brain interstitial fluid (Scheme 1c,d) [26]; (2) a stretchable flexible substrate that conformally fits the skull contour and matches the rabbit parietal curved surface for stable in vivo sampling; and (3) a flocculent Ti_3_AlC_2_-based 2D material with a high specific surface area constructed via NaOH etching and CTAB surfactant-driven self-assembly, which enhances metabolite enrichment and laser desorption/ionization efficiency. We hypothesized that this integrated design would enable in situ capture, highly sensitive detection, and spatial distribution analysis of metabolites in AD-affected brain regions.

Therefore, adopting a structured, hypothesis-driven approach as recommended in recent methodological paradigms [27], this study pursues three specific objectives: (i) to verify the material’s mechanical flexibility (Young’s modulus 0.92 MPa) and anti-interference SALDI-MS performance; (ii) to evaluate the diagnostic value of a multi-marker serum panel (e.g., arachidonic acid) via OPLS-DA; and (iii) most critically, to test the spatial hypothesis—whether arachidonic acid exhibits a unique ‘center-aggregated with peripheral gradient diffusion’ pattern in the AD parietal lobe, providing direct spatial evidence for lipid-targeted interventions.

## 2. Materials and Methods

### 2.1. Reagents and Instruments

Ti_3_AlC_2_ powder (purity ≥98%), sodium hydroxide (NaOH), cetyltrimethylammonium bromide (CTAB), glucose standards, and arachidonic acid standards were of analytical grade and purchased from Sinopharm Chemical Reagent Co., Ltd. (Shanghai 200002, China). Key instruments included a field-emission scanning electron microscope (SEM, Hitachi SU8010, Tokyo, Japan), transmission electron microscope (TEM, JEOL JEM2100F), surface-assisted laser desorption/ionization time-of-flight mass spectrometer (SALDI-TOF MS, Bruker Autoflex Speed, Bruker, Billerica, MA, USA), high-speed centrifuge (Eppendorf 5424R), and constant temperature drying oven. Deionized water was used throughout.

### 2.2. Alkaline Etching of Ti_3_AlC_2_ Substrate

The Ti_3_AlC_2_ powder was dispersed in 1 mol/L NaOH solution, transferred to a high-pressure autoclave, and reacted at 180 °C for 6 h [28,29,30,31]. The product was collected by centrifugation at 8000 rpm for 10 min, washed repeatedly with deionized water until neutral, and vacuum-dried at 60 °C for 12 h to obtain alkaline-etched layered Ti_3_AlC_2_.

### 2.3. CTAB Surfactant Self-Assembly Modification

The alkaline-etched Ti_3_AlC_2_ was dispersed in an aqueous solution containing 1.0 mmol/L CTAB. After ultrasonication for 30 min, the mixture was transferred to a high-pressure autoclave and reacted at 120 °C for 4 h. The product was centrifuged, washed alternately with deionized water and absolute ethanol three times, and vacuum-dried at 60 °C for 8 h to obtain the flocculent Ti_3_AlC_2_-based 2D material [32,33,34].

### 2.4. Preparation of Flexible Microneedle Patch

The synthesized flocculent Ti_3_AlC_2_-based 2D material was uniformly coated onto the surface of a pre-fabricated flexible polydimethylsiloxane (PDMS) substrate (thickness of approximately 200 μm) [35,36,37,38,39]. Petal-like microneedle arrays were constructed using a template-assisted method. Specifically, the microneedle height is 80 μm, the spacing is 95 μm, and the base diameter is 35 μm. After curing at 60 °C for 4 h, the flexible petal-like Ti_3_AlC_2_-based 2D material microneedle patch was obtained.

### 2.5. Structural Characterization, Mechanical and Biocompatibility Characterization

The microscopic morphologies of the alkaline-etched Ti_3_AlC_2_, the CTAB-modified flocculent material, and the microneedle arrays were characterized by SEM and TEM. Elemental composition and distribution were analyzed by energy-dispersive X-ray spectroscopy (EDS). Surface hydrophilicity was assessed using a contact angle goniometer.

The Young’s modulus of the MXene-MNs-SALDI patch was measured using a universal tensile testing machine. The patch was fixed between grips and subjected to a constant-rate tensile load; the stress–strain curve was recorded, and the modulus value was calculated. Meanwhile, the deformation behavior of the patch under uniaxial stretching was observed by optical microscopy, and the tensile strain was recorded.

To evaluate the tissue penetration capability and biocompatibility of the patch, the patch was pressed onto the surface of excised porcine skin and live rabbit skin for 5 min. After removal, the skin samples were processed for hematoxylin and eosin (H&E) staining to assess structural integrity and inflammatory response.

### 2.6. Animal Model, In Vivo Sampling, and Human AD Serum Collection

Male New Zealand rabbits (approximately 2.5 kg) were purchased from Shanghai Experimental Animal Center. The AD rabbit model was established by intraperitoneal co-injection of D-galactose (60 mg/kg) and AlCl_3_ (10 mg/kg) daily for 3 weeks [40,41]. All animal procedures were approved by the ethics committee. For in vivo sampling, rabbits were anesthetized with pentobarbital sodium (40 mg/kg) via the ear vein, and a craniotomy was performed to expose the parietal cortex. The flexible microneedle patch was applied to the parietal region for 5 min. After removal, the patch surface was gently rinsed with deionized water and analyzed directly via SALDI-MS.

For human serum collection, fasting blood samples (5 mL each) were drawn from 15 AD patients and 15 healthy controls via the median cubital vein. The blood was left to clot at room temperature for 30 min, and then centrifuged at 3000 rpm for 15 min at 4 °C. The supernatant serum was aliquoted (200 μL/tube) and stored at −80 °C under strictly controlled temperature conditions. For metabolomic profiling, the serum samples were mixed directly with the composite matrix. The human study protocol was approved under reference number NFEC-202503-JS-NT-3.

### 2.7. SALDI-MS Detection and Metabolomic Analysis

The microneedle patch containing captured metabolites was placed on the SALDI-MS target plate and analyzed using a nitrogen laser (337 nm). The parameters were: acceleration voltage of 20 kV, pulsed extraction delay of 100 ns, and 500 laser shots accumulated per spectrum. The mass spectra were acquired in the range of *m*/*z* 50–1000. Each sample was analyzed in triplicate.

After baseline correction, peak alignment, and normalization, the mass spectrometry data were analyzed using SIMCA software (v14.1) for PCA and OPLS-DA. Differential metabolites were screened using variable importance in projection (VIP > 1.0) and Student’s *t*-test (*p* < 0.05). Pathway enrichment was performed using MetaboAnalyst (Version 5.0, McGill University). Receiver operating characteristic (ROC) curves and the area under the curve (AUC) were generated via GraphPad Prism (Version 8.0.2, GraphPad Software, San Diego, CA, USA).

### 2.8. Spatial Distribution Analysis

To evaluate the spatial distribution of metabolites in the parietal region, the sampling area of the patch was divided into a center zone and a peripheral zone. SALDI-MS detection was performed on each zone, and the relative signal intensity ratio (center/periphery, C/P) was calculated for each metabolite.

## 3. Results

### 3.1. Material Preparation and Morphological Characterization

**Figure 1:**
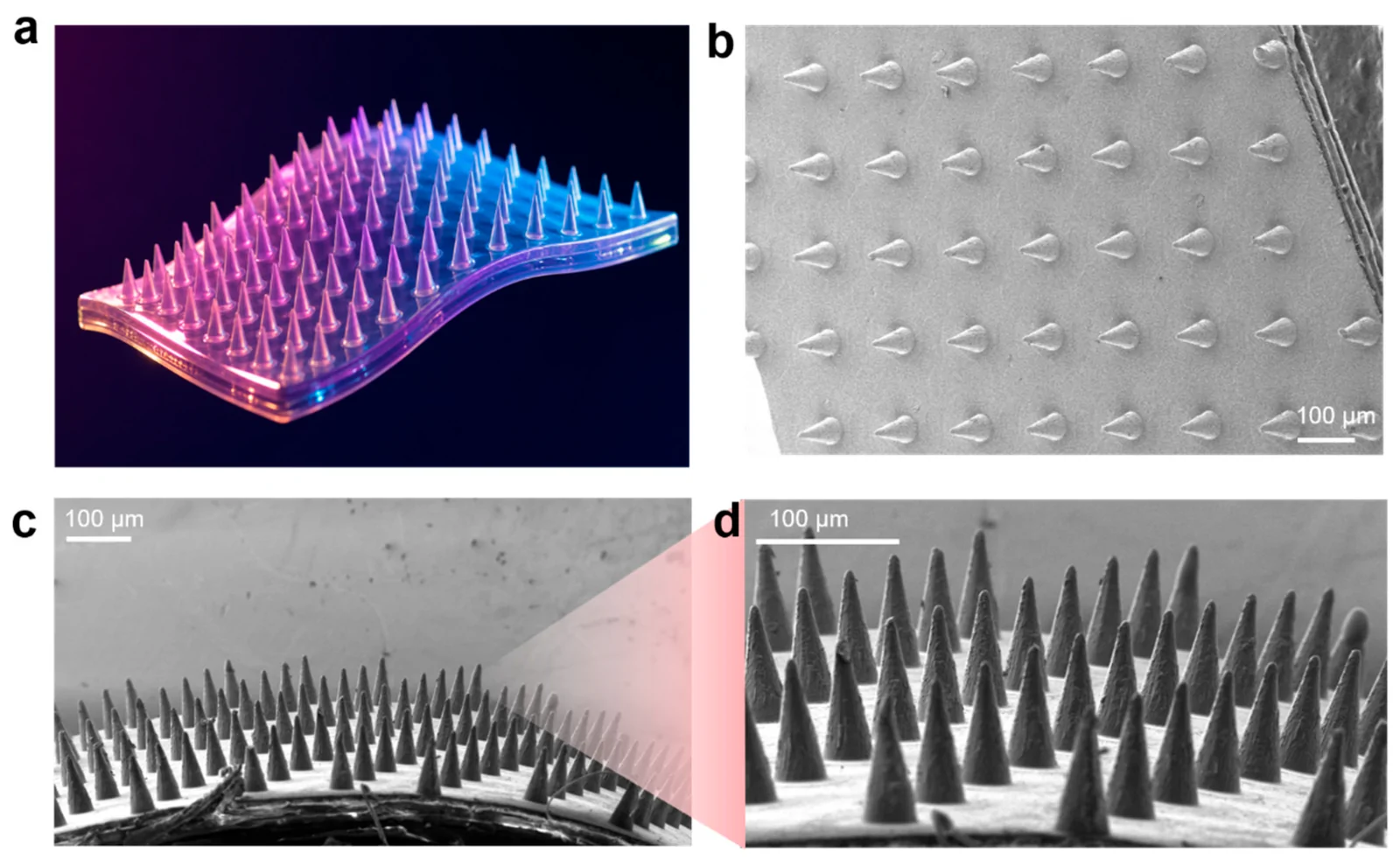
Structural characterization of Ti_3_AlC_2_-based petal-like microneedle arrays. (**a**) Three-dimensional schematic of the petal-like microneedle array. (**b**) Front view showing the uniform distribution of the microneedles. (**c**) Side-view morphology showing the bendability of the flexible substrate. (**d**) Magnified view of a single petal-like microneedle tip, displaying the fine microstructure constructed of the flocculent 2D material.

The overall structure of the Ti_3_AlC_2_-based petal-like microneedle array is characterized (Figure 1). Specifically, the three-dimensional schematic displays the regularly arranged petal-like microneedle array (Figure 1a). The front view shows the uniform distribution of the microneedles (Figure 1b). The side-view morphology demonstrates that the device can undergo free bending deformation based on its highly elastic flexible substrate to conformally fit the curved contour of the cerebral parietal lobe (Figure 1c). Furthermore, the magnified view of a single petal-like microneedle reveals the fine microstructure of the needle tip constructed from the flocculent 2D material (Figure 1d).

**Figure 2:**
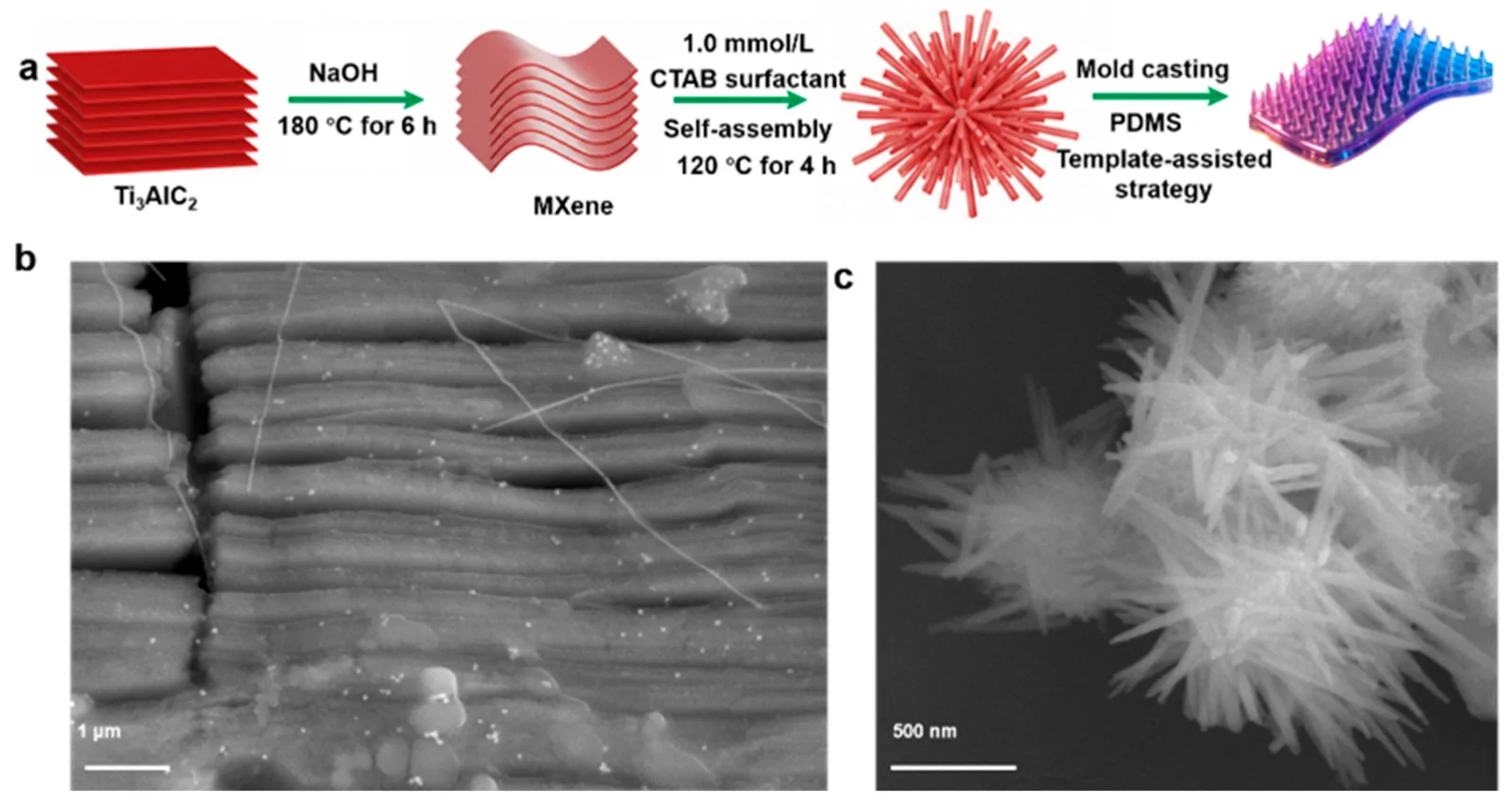
Synthesis process and microscopic morphology of the Ti_3_AlC_2_-based 2D functional material. (**a**) Schematic of the preparation workflow showing NaOH etching followed by CTAB self-assembly and petal-like Ti_3_AlC_2_-based 2D material microneedle patch. (**b**) SEM image of Ti_3_AlC_2_ after one-step NaOH etching, showing a roughened layered structure. (**c**) SEM image of the flocculent Ti3AlC2-based 2D material after CTAB self-assembly, showing a fluffy porous structure.

The synthesis process and electron microscopic morphology of the Ti_3_AlC_2_-based 2D functional material are evaluated (Figure 2). The complete preparation workflow begins with etching the Ti_3_AlC_2_ substrate with NaOH solution, followed by CTAB surfactant-driven self-assembly to yield the flocculent Ti_3_AlC_2_-based 2D material (Figure 2a). The baseline morphology of Ti_3_AlC_2_ after one-step NaOH etching displays a primary roughened layered structure (Figure 2b). After CTAB self-assembly, a fluffy, flocculent, porous microstructure is formed, significantly increasing the specific surface area and surface roughness to provide abundant active sites for metabolite adsorption (Figure 2c). Integrating this material into a microneedle patch, although the microneedles themselves are at the micron scale (80 μm in height, 95 μm in spacing, and 35 μm in base diameter), their internal architecture consists of petal-like MXene nanosheets (Figure 2), which enable efficient enrichment of negatively charged metabolites and sensitive metabolic detection.

### 3.2. Mechanical Properties and In Vivo Conformal Verification

**Figure 3:**
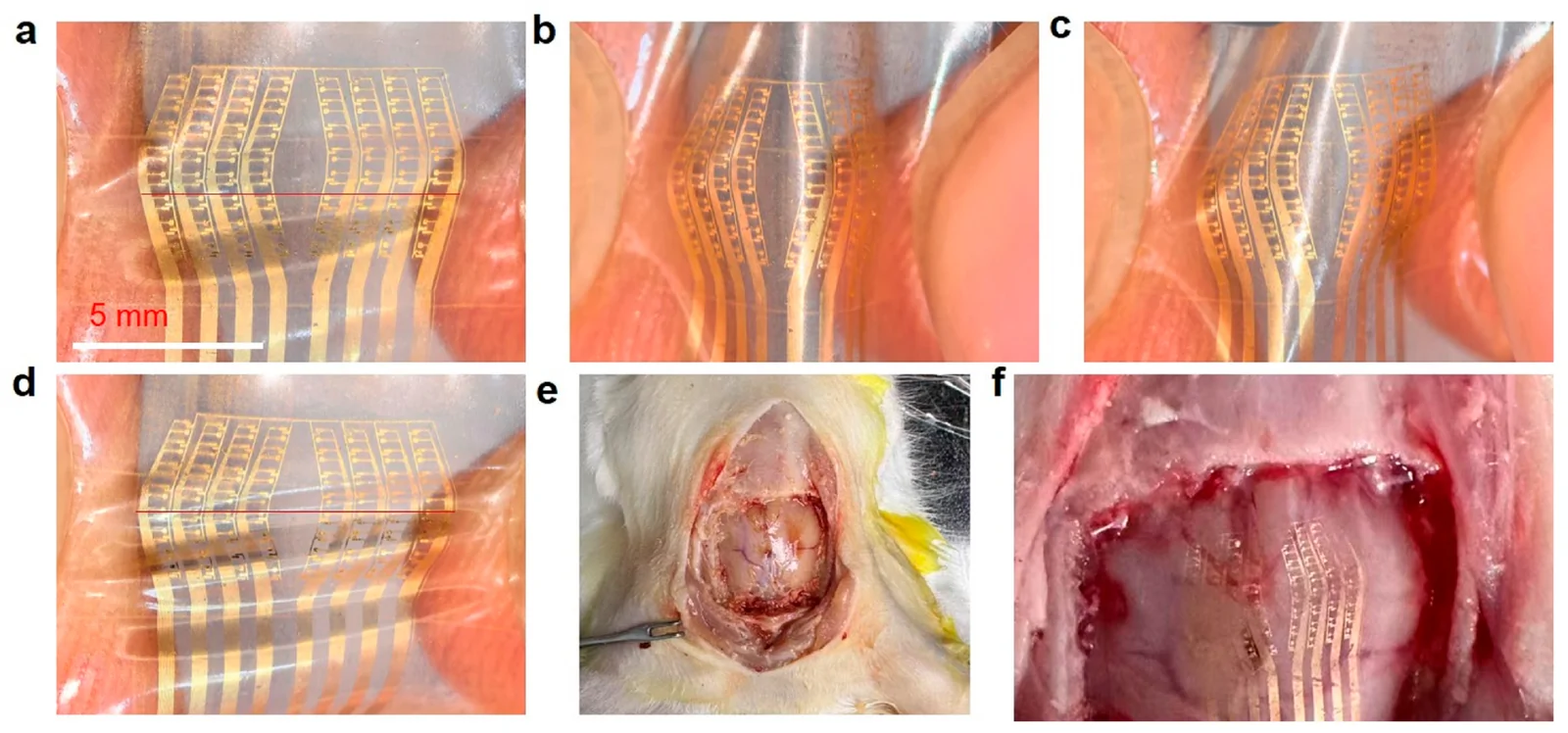
Mechanical properties and in vivo attachment validation of the flexible microneedle patch. (**a**) Schematic of the patch in a flat configuration; (**b**) patch in an arched bending state; (**c**) photograph of large-angle bending deformation; (**d**) patch in a biaxially stretched state (complemented by Figure S1); (**e**) photograph of the craniotomy procedure in an AD rabbit; (**f**) schematic of the patch applied to the parietal region showing good conformal contact.

The mechanical adaptability of the flexible microneedle patch and in vivo experimental characterization in AD rabbits are presented (Figure 3). The flat planar structure of the patch is schematically shown (Figure 3a). The arched, bent state of the patch reflects its outstanding bending performance (Figure 3b). The photograph captures large-angle bending deformation (Figure 3c). The bidirectional stretching state of the patch verifies its high tensile flexibility (Figure 3d and Figure S1). We performed Young’s modulus analysis on our MXene-MNs-SALDI Biopatch, and the measured modulus value was 0.92 MPa (S2), which is on the same order of magnitude as that of biological tissues, with a slight tensile strain of 15.2%.

This ensures that the patch can effectively extract metabolites while avoiding the significant trauma caused by rigid microneedles. Furthermore, we conducted H&E staining analysis after applying the MXene-MNs-SALDI Biopatch to the skin. The results demonstrated that, compared with the control group, the MXene-MNs-SALDI Biopatch did not induce obvious skin inflammation (Figures S3 and S4), and the use of CTAB as a surfactant did not compromise its biocompatibility, indicating that the patch possesses favorable biocompatibility.

These mechanical characterizations confirm that the patch possesses favorable flexibility to adapt to the irregular curved surface of brain tissue. The photograph of the craniotomy in an AD rabbit model shows the fully exposed parietal brain tissue (Figure 3e). Finally, the schematic of the flexible microneedle patch applied to the rabbit parietal lobe demonstrates conformal contact with the brain surface, establishing the physical foundation for in situ metabolite collection (Figure 3f).

### 3.3. Mass Spectrometry Detection Performance

**Figure 4:**
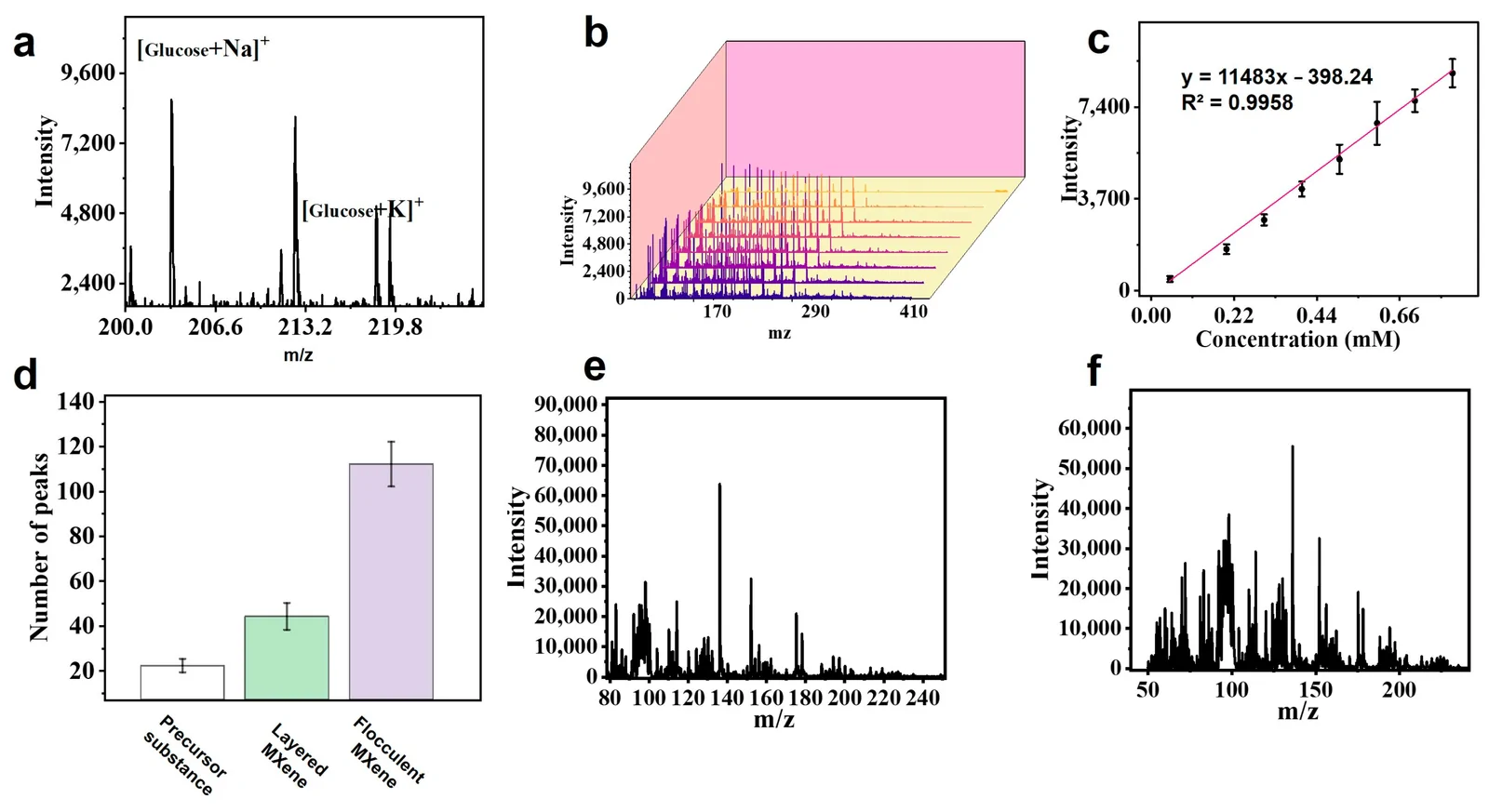
Quantitative detection performance of the MXene-MNs-SALDI-Biopatch. (**a**) Mass spectrum of glucose with characteristic peaks and sodium/potassium adducts (*m*/*z* 203, 219); (**b**) series of mass spectra at gradient glucose concentrations; (**c**) calibration curve for glucose over 0.08–0.8 mmol/L (R^2^ = 0.9958); (**d**) The mass spectrometry signal of the precursor substance was only 1453 ± 112, while that of the layered MXene material was enhanced to 3644 ± 342, and that of the final flocculent MXene material was enhanced to 5763 ± 326. The signals increased by approximately 2.5-fold and 1.6-fold after the two modification steps, respectively; (**e**) salt interference test (150 mM NaCl); (**f**) protein interference test (5% BSA).

The SALDI-MS quantitative performance of the flexible microneedle patch is analyzed (Figure 4). The mass spectrum of glucose obtained using the microneedle patch shows clearly identified characteristic peaks, alongside prominent sodium (*m*/*z* 203) and potassium (*m*/*z* 219) adduct peaks, confirming the favorable signal recognition capability of the device (Figure 4a). The series of mass spectra corresponding to gradient concentrations of glucose exhibit a clear incremental trend in signal intensity with increasing concentration (Figure 4b). The quantitative standard curve of glucose in the concentration range of 0.08–0.8 mmol/L yields a linear correlation coefficient of *R*^2^ = 0.9958, demonstrating a wide and excellent linear detection range (Figure 4c). Considering that CTAB, as a surfactant, can adequately modulate the performance of the MXene-MNs-SALDI Biopatch, it does not interfere with the SALDI-MS performance.

As shown in Figure 4d, the mass spectrometry signal of the precursor substance was only 1453 ± 112, while that of the layered MXene material was enhanced to 3644 ± 342, and that of the final flocculent MXene material was enhanced to 5763 ± 326. The signals increased by approximately 2.5-fold and 1.6-fold after the two modification steps, respectively, demonstrating that the stepwise modification protocol progressively enhances the material’s metabolite enrichment and recognition capabilities.

The salt tolerance and anti-protein interference performance of the device are tested (Figure 4e,f). In the presence of high salt (150 mM NaCl) and high protein (5% BSA), the intensity of the characteristic glucose mass spectrometry peaks does not exhibit significant attenuation, demonstrating that the Ti_3_AlC_2_-based material possesses outstanding resistance to salt and protein interference, making it highly suitable for the direct analysis of complex brain interstitial fluid samples (Figure 4e,f).

### 3.4. Serum Metabolomic Profiling

**Figure 5:**
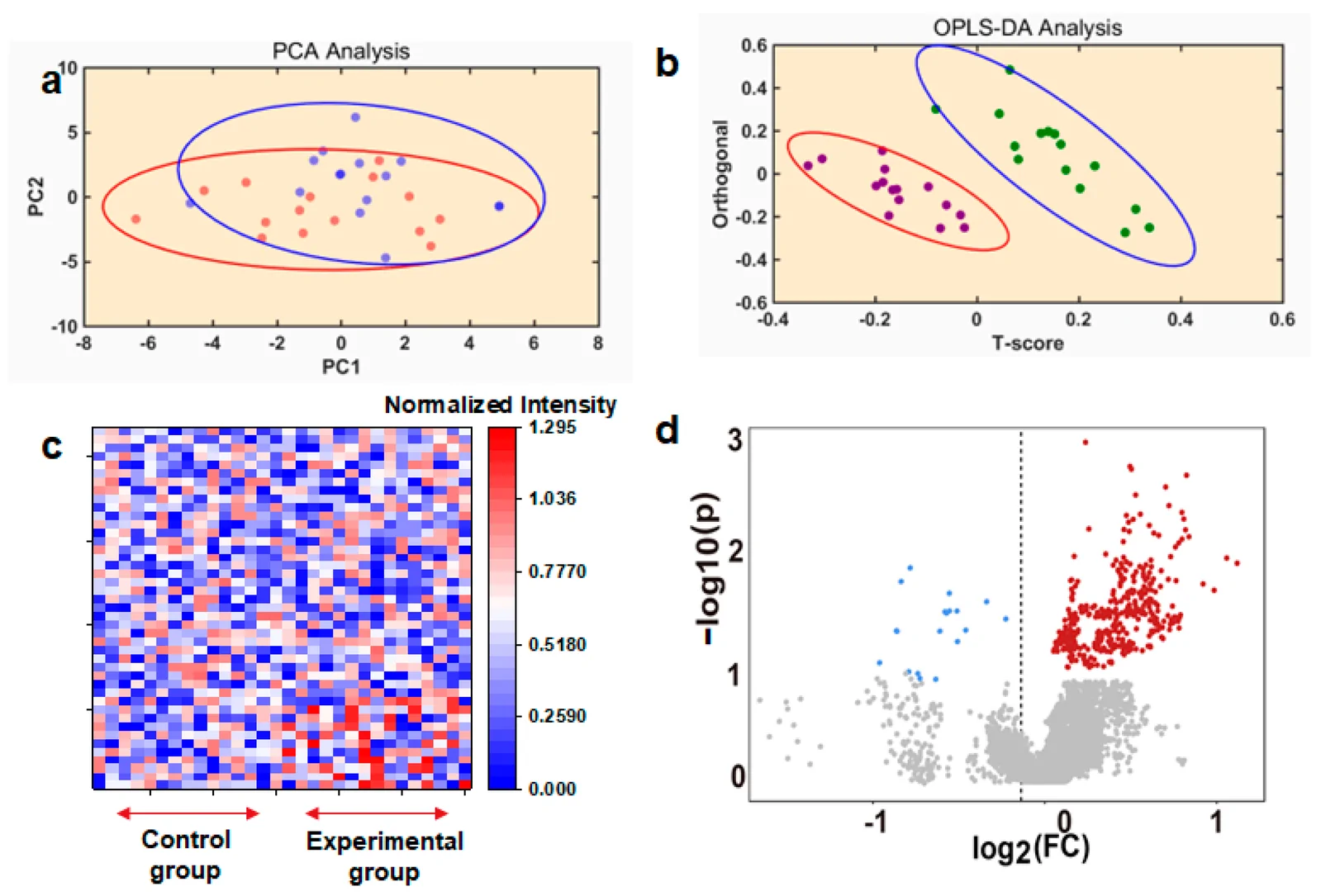
Metabolic profiling analysis of serum samples from AD patients and healthy controls using the MXene-MNs-SALDI-Biopatch. (**a**) PCA score plot showing overlapping distributions; (**b**) OPLS-DA score plot showing clear separation; (**c**) heatmap of differential metabolites; (**d**) volcano plot (VIP > 1.0, *p* < 0.05).

Metabolic profiling analysis of 15 human AD patient samples and 15 healthy controls is performed (Figure 5). Principal component analysis (PCA) shows that the normal and AD samples exhibit overlapping distributions in the 2D score plot, failing to achieve effective separation (Figure 5a). In contrast, the orthogonal partial least squares discriminant analysis (OPLS-DA) score plot reveals a clear boundary between the two groups without overlap, indicating that AD-associated metabolic reprogramming possesses multidimensional and subtle features that supervised learning methods can effectively extract (Figure 5b). A clustering heatmap based on the OPLS-DA results shows significant differences in the levels of various metabolites between the AD and control groups, displaying consistent intra-group and distinct inter-group clustering patterns (Figure 5c). Furthermore, a volcano plot (using VIP > 1.0 and *p* < 0.05 as thresholds) highlights a large number of statistically significant differential metabolic markers, with upregulated metabolites dominated by phospholipid intermediates and downregulated metabolites mainly consisting of amino acids (Figure 5d).

### 3.5. Diagnostic Efficacy and Pathway Enrichment

**Figure 6:**
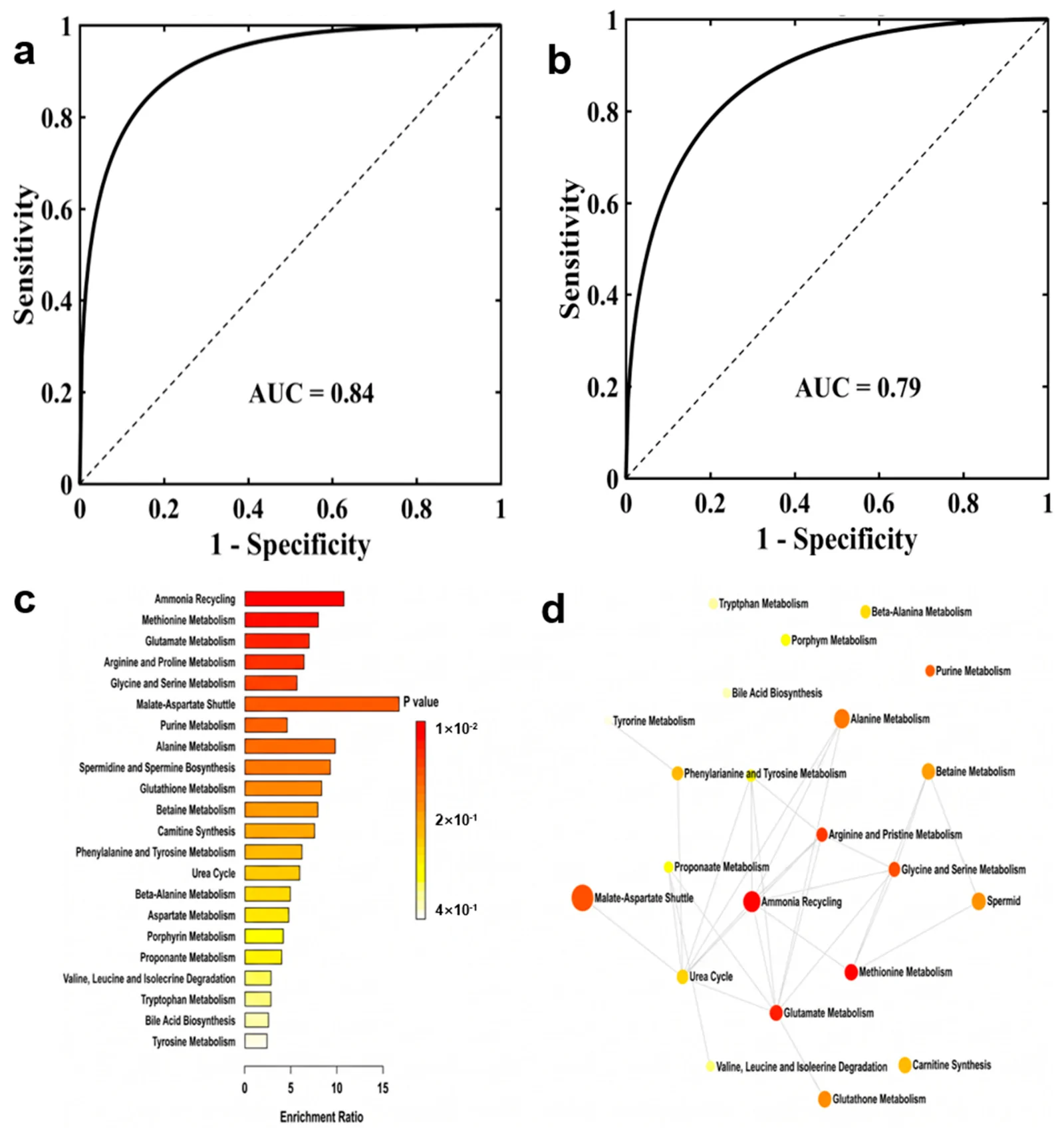
Diagnostic efficacy and pathway enrichment analysis of key metabolic biomarkers. (**a**) ROC curve of the combined diagnosis using the top 5 markers (AUC = 0.84); (**b**) ROC curve of arachidonic acid alone (AUC = 0.79); (**c**) pathway enrichment bubble plot; (**d**) metabolic pathway interaction network.

The diagnostic efficacy and pathway enrichment of key metabolic markers are analyzed (Figure 6). A combined receiver operating characteristic (ROC) curve analysis of the top 5 differential markers—including arachidonic acid, proline, glutamine, phosphocholine, and an unidentified lipid metabolite—yields an area under the curve (AUC) of 0.84, with a sensitivity of 0.89 and specificity of 0.70 (Figure 6a). Individually, the core biomarker arachidonic acid achieves a diagnostic AUC of 0.79 (Figure 6b), while proline yields an AUC of 0.73 (Figure S6), suggesting that single metabolic markers offer limited diagnostic efficacy, whereas multi-marker models significantly enhance diagnostic performance (Figure 6b and Figure S6).

Pathway linkage analysis of all differential metabolites revealed significant enrichment in multiple pathways, including arachidonic acid metabolism, glycerophospholipid metabolism, alanine/aspartate/glutamate metabolism, and tryptophan metabolism (Figure 6c). Among these, the significantly altered metabolites comprised arachidonic acid, glycine, phenylalanine, tryptophan, and methionine (VIP > 1.0 and *p* < 0.05, as the screening thresholds). The *t*-test results indicated that arachidonic acid levels were significantly different between the control group and the Alzheimer’s disease group (Figure S5), with *p* < 0.01. In addition, we performed a recovery test for arachidonic acid (n = 5), which yielded recovery rates ranging from 98.2% to 103.2% (Table S1), with a relative standard deviation (RSD) of 2.01%, indicating satisfactory precision. In addition, we have performed a comparative analysis of our MXene-MNs-SALDI strategy with current state-of-the-art techniques (Table S2) [42,43]. Our results demonstrate that the proposed MXene-MNs-SALDI approach exhibits overall superior performance in certain aspects.

The interaction network of these metabolic pathways demonstrates close regulatory connections among different pathways, suggesting that AD-associated parietal lesions involve the reprogramming of complex, multi-pathway metabolic networks, with lipid metabolism pathways situated at the core node of the network (Figure 6d).

### 3.6. Spatial Distribution of Parietal Metabolites

**Figure 7:**
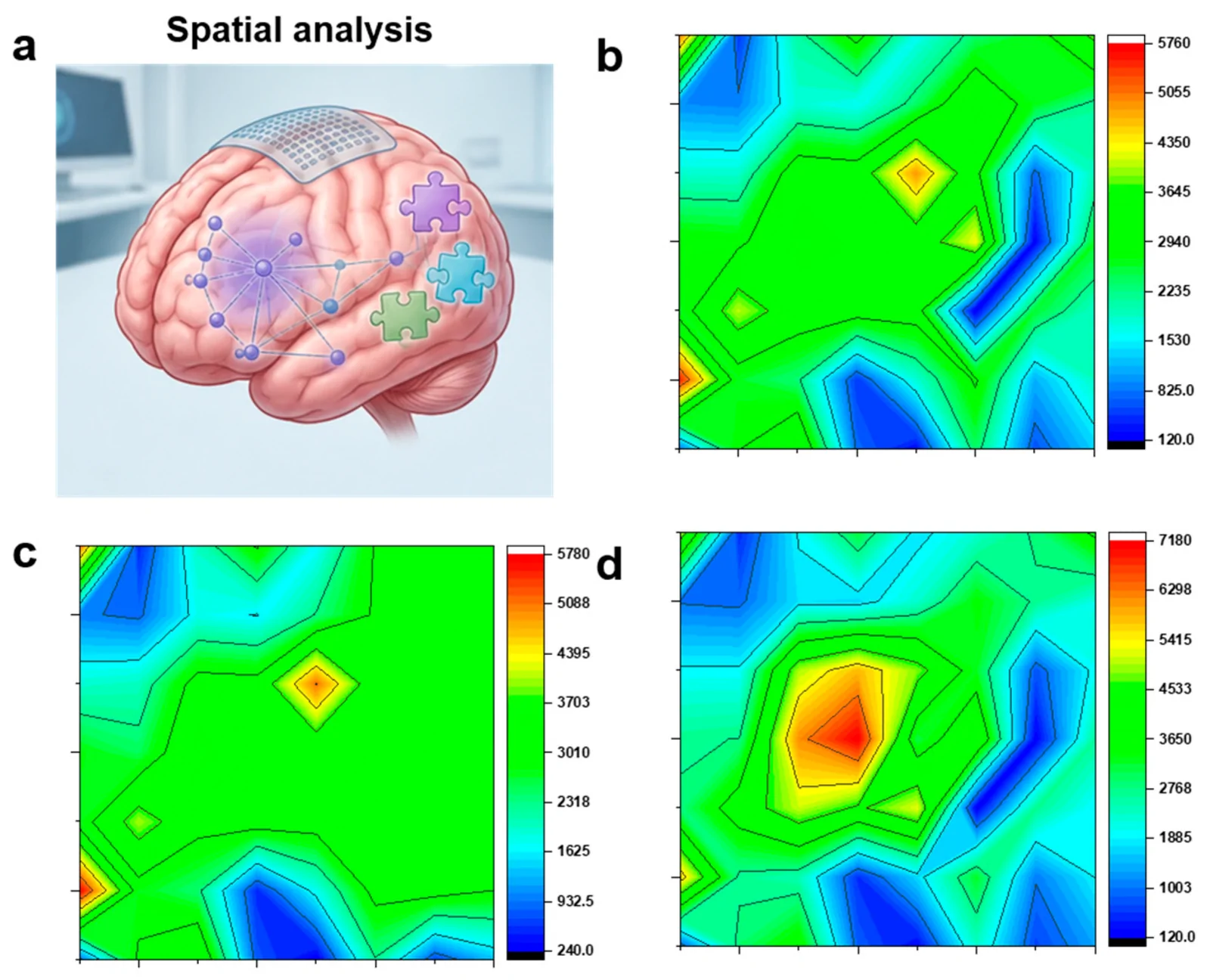
Spatial distribution characteristics of parietal lobe metabolites analyzed by the MXene-MNs-SALDI-Biopatch. (**a**) Schematic of in situ sampling on the rabbit parietal lobe; (**b**,**c**) representative non-aggregating metabolites; (**d**) spatial distribution of arachidonic acid showing center-convergence with peripheral gradient-decreasing diffusion, with C/P ratio significantly greater than 1.5 (*p* < 0.01).

The spatial distribution of metabolites within the sampling area of the flexible microneedle patch is analyzed (Figure 7). In situ sampling is conducted on the parietal cortex of New Zealand rabbits (Figure 7a), and the collected metabolites are quantified for signal intensities in the center and peripheral regions. Most metabolic products exhibit a center-to-periphery (C/P) signal intensity ratio close to 1.0, showing no significant global aggregation or dispersion across the parietal region (Figure 7b,c). In contrast, arachidonic acid displays a unique spatial distribution characterized by a gradient decline in signal intensity from the center toward the periphery, representing a localized aggregation pattern (Figure 7d). The corresponding C/P ratio is significantly greater than 1.5 (*p* < 0.01) (Figure 7d). This specific spatial pattern indicates that arachidonic acid and its metabolic pathway may play a distinct regional regulatory role in AD parietal pathology, and its localized aggregation might be associated with focal activation of AD-related neuroinflammation or lipid peroxidation (Figure 7d).

## 4. Discussion

### 4.1. Design Advantages and Detection Mechanism of the Flexible Microneedle Patch

In this study, a petal-like Ti_3_AlC_2_-based two-dimensional flexible microneedle patch was fabricated, and its SALDI-MS detection performance was systematically evaluated. Compared with the material modified solely by alkaline etching, the flocculent Ti_3_AlC_2_-based material functionalized via CTAB self-assembly increased the intensity of identifiable metabolite, demonstrating significant improvements in both signal intensity and metabolite coverage (Figure 4d). This enhancement can be attributed to two synergistic effects: first, alkaline etching introduces micro-/nano-roughness and oxygen-containing functional groups onto the Ti_3_AlC_2_ surface, increasing the density of surface active sites; second, CTAB, as a cationic surfactant, drives electrostatic self-assembly to form a fluffy porous structure, which expands the specific surface area and enhances surface hydrophobicity, thereby promoting metabolite enrichment and energy transfer during laser desorption/ionization.

Furthermore, the flexible microneedle patch retains stable detection signals under high-salt (150 mM NaCl) and high-protein (5% BSA) conditions, demonstrating excellent anti-matrix interference capabilities (Figure 4e,f). This resistance is attributed to the intrinsic chemical stability of the Ti_3_AlC_2_-based composite and the physical exclusion of inorganic salts and large proteins by the porous micro-/nanostructure. The micro-/nanopores permit the rapid diffusion of small-molecule metabolites to the active surface while excluding macromolecular proteins from competitive absorption. Compared to existing carbon- or noble-metal-based SALDI matrices, the synthesized flocculent Ti_3_AlC_2_ material offers comprehensive advantages in cost, biocompatibility, and anti-interference capacity, providing a reliable platform for the direct analysis of living brain metabolites.

### 4.2. Metabolomic Profiling and Diagnostic Significance of Differential Metabolites

In this study, conventional unsupervised PCA fails to differentiate the serum metabolic profiles of AD patients from healthy controls (Figure 5a), whereas supervised OPLS-DA achieves distinct separation between the two groups (Figure 5b). This indicates that AD-associated metabolic reprogramming possesses multidimensional and subtle features that supervised learning methods can effectively extract. Multiple significantly differential metabolites are identified via the heatmap and volcano plot, among which arachidonic acid (AUC = 0.79) and proline (AUC = 0.73) show moderate diagnostic potential as single markers (Figure 5c,d). These findings underscore that single metabolic markers offer limited diagnostic efficacy, reflecting the complexity and heterogeneity of metabolic regulation in neurodegenerative diseases.

Pathway enrichment analysis reveals that the differential metabolites are predominantly enriched in arachidonic acid metabolism, glycerophospholipid metabolism, and glutamate metabolism pathways (Figure 6c). Arachidonic acid is a key component of cell membrane phospholipids, and its metabolism involves the synthesis of inflammatory mediators (such as prostaglandins and leukotrienes) mediated by cyclooxygenases and lipoxygenases. During AD pathogenesis, *β*-amyloid deposition and neuroinflammatory responses can activate phospholipase A_2_, leading to membrane phospholipid hydrolysis and the release of arachidonic acid, which cascades into inflammatory responses. The localized aggregation of arachidonic acid observed in the parietal region of the AD model is highly consistent with this pathological mechanism (Figure 7d), confirming the central role of lipid metabolic disorders in AD.

### 4.3. Innovation and Significance of Arachidonic Acid Spatial Aggregation

**Figure 8:**
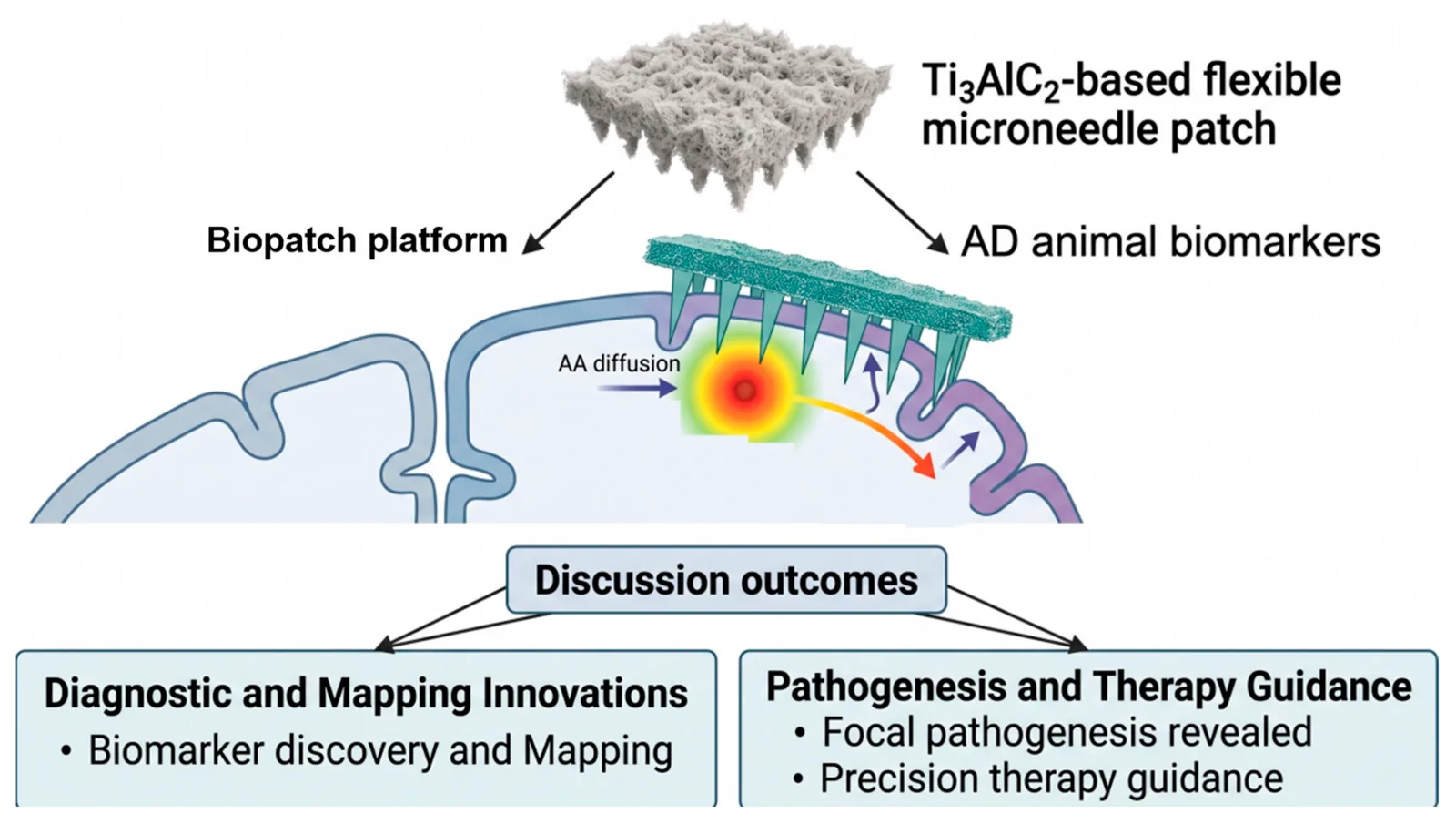
The MXene-MNs-SALDI Biopatch was applied for the spatiotemporal analysis of arachidonic acid in Alzheimer’s disease.

The core innovation of this study lies in the elucidation of the unique spatial distribution pattern of arachidonic acid at the living parietal lobe level, characterized by centralized aggregation with peripheral diffusion (Figure 8).

This finding is significant in three respects: first, it complements the spatial dimension of metabolomic information. Traditional studies typically report only overall concentration changes, overlooking tissue heterogeneity, whereas our results show that even within the same brain region, metabolite distribution is non-uniform, with arachidonic acid forming localized hotspots that may correspond to initiation sites or active lesion zones of AD pathology, thereby offering a new perspective on focal pathogenesis; second, the focal aggregation pattern implies that lipid metabolic disorders in the parietal lobe do not arise as diffuse global alterations, but rather originate from a local core and spread outward, a “center-to-periphery” propagation that conceptually mirrors the prion-like transmission hypothesis of pathological proteins (Aβ, tau), with the aggregation zone potentially serving as the epicenter of lipid peroxidation and neuroinflammation from which downstream inflammatory mediators (e.g., isoprostanes, leukotrienes) diffuse to drive broader neuronal dysfunction; and third, this spatial pattern provides guidance for targeted therapeutic intervention, as key enzymes in the arachidonic acid pathway (e.g., cytosolic phospholipase A_2_ and cyclooxygenase-2) are likely highly expressed or activated within the aggregation zone, suggesting that future strategies could exploit local lipid metabolism modulation—through focal delivery of specific inhibitors or nanomedicines—to achieve precision therapy for AD while minimizing systemic side effects.

### 4.4. Study Limitations

Several limitations in the current study should be acknowledged: (1) the pathological characteristics of the rabbit AD model (Aβ deposition and neurofibrillary tangles) differ to some extent from human AD, requiring further validation in transgenic AD mice or other models closely resembling human pathology; (2) the spatial sampling partition is relatively coarse, warranting the future use of mass spectrometry imaging with higher spatial resolution or array-type multi-channel microneedles to construct finer two-dimensional spatial maps; (3) the current 5 min single-sampling approach lacks dynamic monitoring capabilities, highlighting the need for repeatable or continuous sampling strategies to track the spatiotemporal evolution of metabolites along with AD progression; (4) due to the small sample size (*n* = 15), the diagnostic efficacy of the combined multi-marker model (AUC = 0.84) must be validated in independent, larger cohorts.

## 5. Conclusions

A petal-like Ti_3_AlC_2_-based 2D material flexible microneedle patch was successfully constructed. Through NaOH etching and CTAB self-assembly, a flocculent, porous structure with a high specific surface area was created, enhancing metabolite adsorption and SALDI-MS detection performance. The patch exhibits excellent mechanical flexibility, salt/protein tolerance, and a wide linear detection range, allowing conformal attachment to the rabbit parietal cortex for in situ metabolite extraction. In parietal metabolic profiling, OPLS-DA achieved clear separation between the AD and control groups, and the multi-marker diagnostic model (AUC = 0.84) outperformed single markers. Crucially, this study first reveals the unique spatial aggregation of arachidonic acid in the AD parietal region, characterized by a centralized gathering and a gradient peripheral diffusion pattern. This finding provides spatial evidence supporting the lipid metabolic reprogramming hypothesis in AD and suggests new avenues for targeted localized interventions.

## Data Availability

The raw data supporting the conclusions of this article will be made available by the authors on request.

## Supplementary Materials

The following supporting information can be downloaded at: https://www.mdpi.com/article/10.3390/molecules31203665/s1, Figure S1: Biopatch in a biaxially stretched state; Figure S2: Young’s modulus analysis; Figure S3: H&E staining analysis; Figure S4: Photograph of in vivo penetration; Figure S5: *T*-test analysis of arachidonic acid; Figure S6: ROC curve of proline; Table S1: Spike-and-recovery analysis for arachidonic acid; Table S2: The comparison between the MXene-MNs-SALDI and other platforms.
