# Supplementary material for: Flocculent Ti3AlC2-Based Two-Dimensional Material Flexible Microneedle Patch for Screening Alzheimer’s Disease Animal Biomarkers and In Situ Revealing Spatial Aggregation of Arachidonic Acid
Source: Molecules. 2026 Oct 13;31(20):3665. doi: 10.3390/molecules31203665 (PMC13707684; doi:10.3390/molecules31203665)
Supplement: Supplementary file 1 [file molecules-31-03665-s001.zip › molecules-4482078-supplementary.pdf]

Flocculent Ti_3AlC_2-Based Two-Dimensional Material Flexible Microneedle Patch for Screening Alzheimer's Disease Animal Biomarkers and In Situ Revealing Spatial Aggregation of Arachidonic Acid

Siyuan Chen [1], Bixiang Li [2], Tangjing Zhao [1], Qin Huang [1], Chengyu Li [1], Yilamujiang Ainiwan [1], Songtao Qi [1,*] and Jun Pan [1,*]

[1] Department of Neurosurgery, Nanfang Hospital, Southern Medical University, Guangzhou 510515, China; wgrasssfish@163.com (S.C.); tangjingzhao7713@163.com (T.Z.); huangqin67@outlook.com (Q.H.); chengyuli4967@163.com (C.L.); yilamujiang6809@163.com (Y.A.)

[2] Department of Neurology, Zhujiang Hospital, Southern Medical University, Guangzhou 510515, China; icarusforever@163.com

* Correspondence: qisongtaosjwk@163.com (S.Q.); panjundu@smu.edu.cn (J.P.)

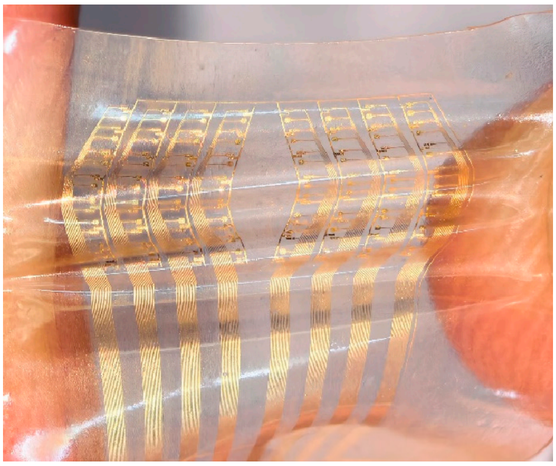

Figure S1. Biopatch in a biaxially stretched state.

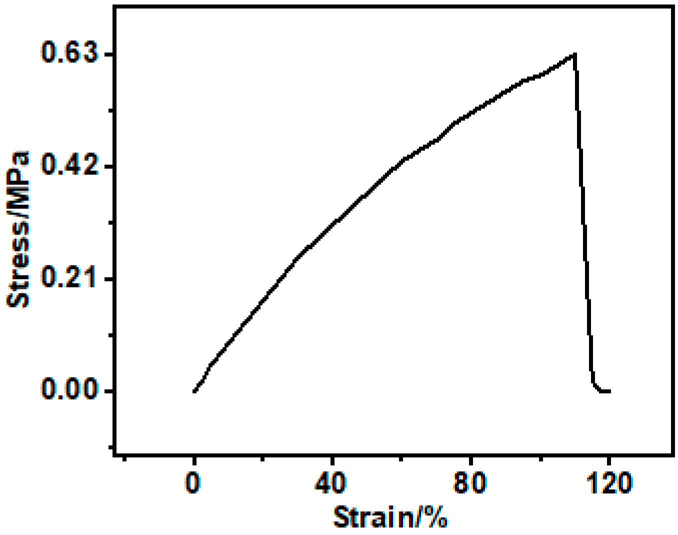

Figure S2. Young's modulus analysis of the MXene-MNs-SALDI Biopatch shows a value of 0.92 MPa.

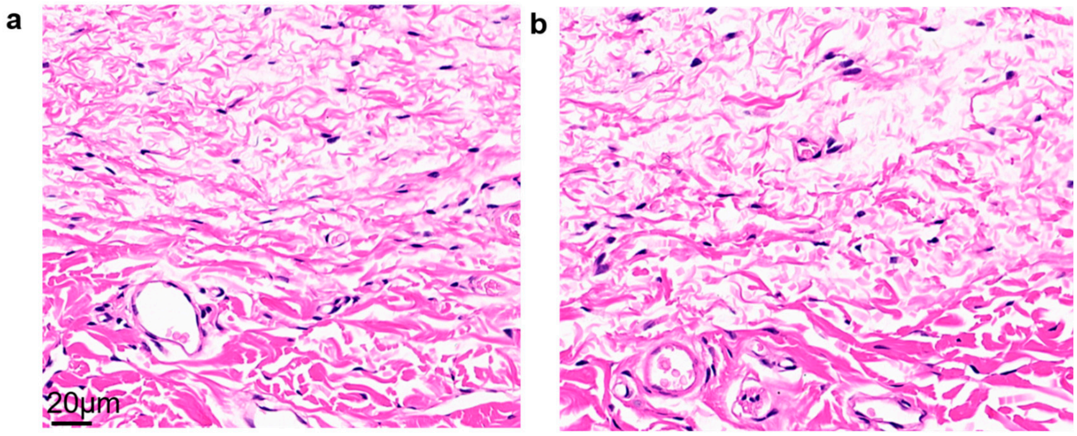

Figure **S3.** H&E staining analysis: a) H&E staining results of the control group skin; b) H&E staining analysis performed 5 minutes after the application of the MXene-MNs-SALDI Biopatch. The application of the MXene-MNs-SALDI Biopatch did not cause any obvious infection to the skin.

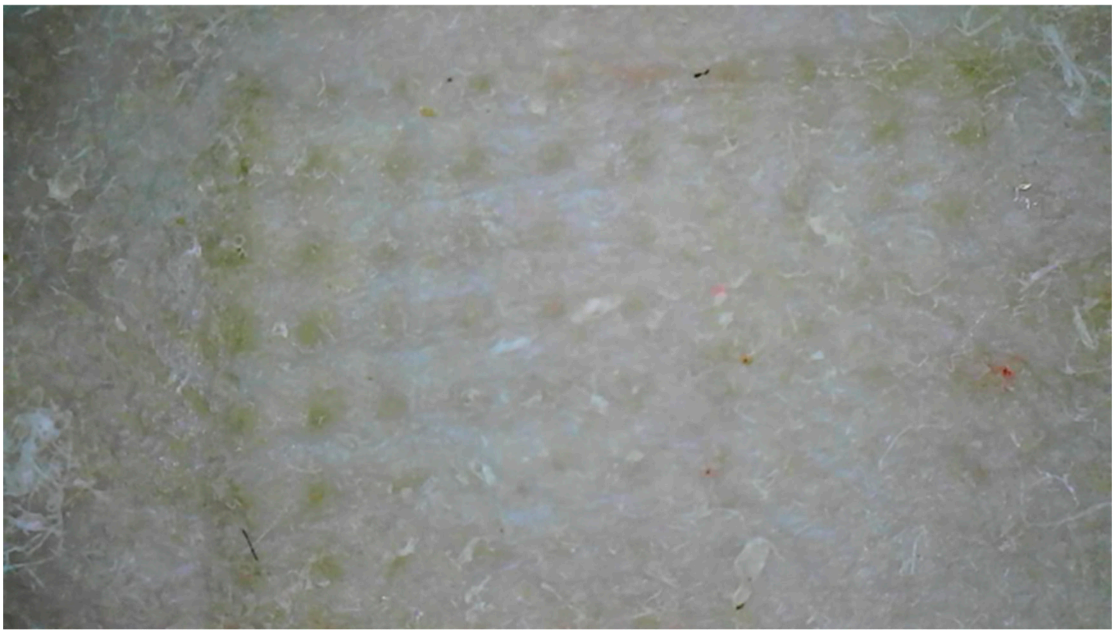

Figure **S4.** Photograph of the MXene MNs SALDI Biopatch applied to/penetrating rabbit skin in vivo.

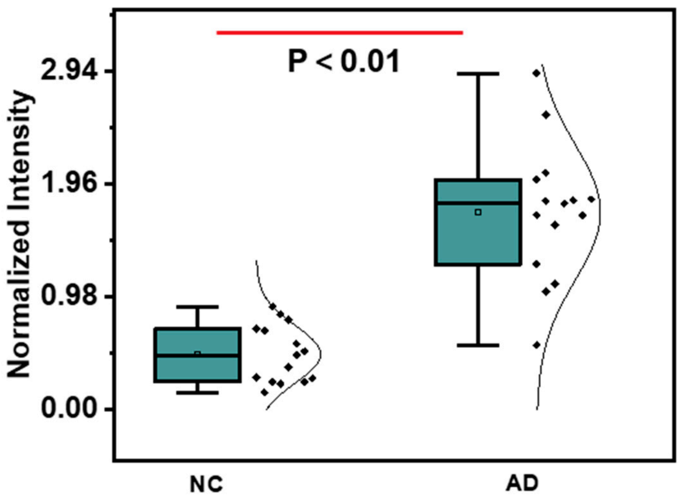

Figure **S5.** Comparison of arachidonic-acid signals in serum from 15 patients with Alzheimer’s disease and 15 healthy controls.

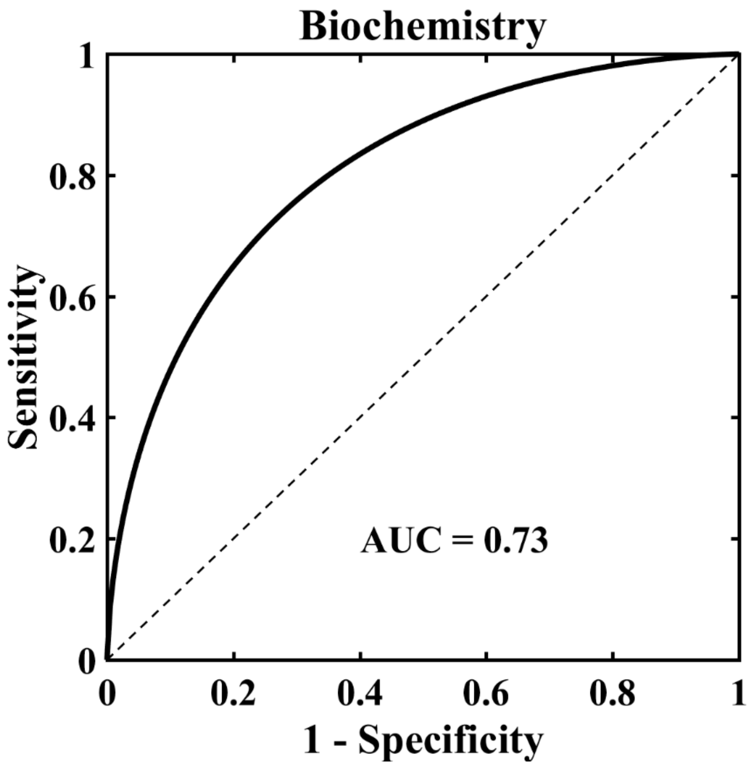

Figure **S6.** Proline yields an AUC of 0.73.

Table S1. spike-and-recovery analysis for arachidonic acid. (Supporting information)

Spike solution number	Arachidonic acid (%)	Spiked recovery (%)
1	100	103.2
2	100	99.5
3	100	102.2
4	100	98.2
5	100	101.4
Average (n=5)		100.88
SD		2.02
RSD		2.01

Table S2. The comparison between the MXene-MNs-SALDI and other platforms.

Number	Platform	Throughput	In situ or not	AUC value	Spatial analysis capability	Reference
1	Electrochemical platform	No	No	0.84	No	(Arjun et al., 2025)
2	*N*-Glycans	Yes	No	0.86	No	(Onigbinde et al., 2025)
3	MXene-MNs-SALDI	Yes	Yes	0.860.84	Yes	This work
